# Extraction, Isolation of Bioactive Compounds and Therapeutic Potential of Rapeseed (*Brassica napus* L.)

**DOI:** 10.3390/molecules27248824

**Published:** 2022-12-12

**Authors:** Nazym Tileuberdi, Aknur Turgumbayeva, Balakyz Yeskaliyeva, Lazzat Sarsenova, Raushan Issayeva

**Affiliations:** 1 Faculty of Medicine and Healthcare, Higher School of Medicine, Al-Farabi Kazakh National University, Almaty 050040, Kazakhstan; 2 Faculty of Chemistry and Chemical Technology, Al-Farabi Kazakh National University, Almaty 050040, Kazakhstan

**Keywords:** *Brassica napus* L., extraction methods, isolation, rapeseed oil, biocompounds, antioxidant properties, pharmacological activity

## Abstract

Rapeseed (*Brassica napus* L.) is a herbaceous annual plant of the Cruciferous family, the Cabbage genus. This oilseed crop is widely used in many areas of industry and agriculture. High-quality oil obtained from rapeseed can be found in many industrial food products. To date, extracts with a high content of biologically active substances are obtained from rapeseed using modern extraction methods. *Brassica napus* L. seeds contain polyunsaturated and monounsaturated fatty acids, carotenoids, phytosterols, flavonoids, vitamins, glucosinolates and microelements. The data in this review show that rapeseed biocompounds have therapeutic effects in the treatment of various types of diseases. Some studies indicate that rapeseed can be used as an anti-inflammatory, antioxidant, antiviral, hypoglycemic and anticancer agent. In the pharmaceutical industry, using rapeseed as an active ingredient may help to develop new forms drugs with wide range of therapeutic effects. This review focuses on aspects of the extraction of biocompounds from rapeseed and the study of its pharmacological properties.

## 1. Introduction

Rapeseed (*Brassica napus* L.) is an amphidiploid resulting from interspecific hybridization between kale (*Brassica oleracea* L.) and turnip (*Brassica rapa*) [1]. There are two varieties of rapeseed: spring and winter. Spring rapeseed usually does not require a vernalization period like winter rapeseed and is grown where environmental conditions in winter are not conducive to the survival of rapeseed plants. Winter rapeseed has a more powerful root system than spring rapeseed, which is associated with a higher seed yield [2].

Rapeseed is cultivated in large volumes in China, Canada, the USA, India, European countries, Russia and Kazakhstan. In Kazakhstan, rapeseed is grown mainly in the regions of North Kazakhstan, Kostanay, Turkestan and Almaty [3]. Rapeseed in Kazakhstan began to spread from the beginning of 2000 as one of the important areas of production diversification, supported by state subsidies, and firmly entered the structure of crop rotations in the northern regions of Kazakhstan. The annual area of spring rapeseed in Kazakhstan is 150–350 thousand hectares and rapeseed oilseeds have become an export product that is in stable demand [4]. *Brassica napus* L. has great potential for agro-industrial development and is therefore a major target for crop improvement. To this end, researchers and breeders are working diligently to develop new varieties of rapeseed with improved agronomic characteristics such as disease resistance, herbicide resistance and increased resistance to certain biotic and abiotic stresses [5].

The significant interest in rapeseed observed all over the world due to the fact that this crop contains up to 49% fat and more than 20% protein, making it a valuable raw material for the oil and fat industry, as well as the feed industry [6]. Gabriella Di Lena et al. provided data on the prospects for the use of rapeseed meal in various industries, as well as on its nutritional properties and contained components [7]. The rapeseed market in Europe is mainly driven by the demand for rapeseed oil, which is mainly used in the food industry and to a lesser extent, in the biodiesel industry sector [8]. In addition, the European strategy for promoting protein crops promotes the cultivation of protein-rich crops, including rapeseed, to reduce dependence on imported vegetable proteins and accelerate the transition to more sustainable agri-food systems. Every part of rapeseed (flowers, seeds, leaves, stem and root) is used for food, medicinal and cosmetic purposes. The seeds are the most important part as they are used as a source of oil and protein. The content of oils and proteins varies in different lines of rapeseed varieties [9]. Rapeseed meal contains proteins with a balanced amino acid composition, so it can be used as an alternative source of protein, thereby satisfying the global demand for protein [10]. Rapeseed meal is characterized by a high fiber content (33–40%), protein content (31–34%) and the presence of carbohydrates (6–13%) and ash (5.5–6.8%). The sum of the concentrations of all detected phenolic compounds is about 400 mg per kg of rapeseed meal. The most abundant phenolic compound in rapeseed meal is sinapic acid, which accounts for over 85% of all quantified phenolic compounds (mean 357 ± 13 mg/kg, range 339–379 mg/kg). Hydroxycinnamic acid derivatives are reported to be the most abundant bioactive compounds in rapeseed, with sinapine having antitumor, neuroprotective, antioxidant and hepatoprotective properties that are important for health [11].

High-quality oil with useful properties is obtained from *Brassica napus* L. seeds. Rapeseed oil is one of the best oils containing omega-3, omega-6 fatty acids, vitamins, phytosterols and terpenes. Rapeseed oil is the third most important vegetable oil in the world after palm and soybean oils [12]. Moreover, due to its valuable ingredients, rapeseed oil is widely used throughout the world as a healthy alternative to olive oil [13]. Rapeseed oil is also widely used in the food, chemical and cosmetic industries [14]. The low amount of saturated fatty acids in rapeseed oil helps to reduce blood cholesterol levels. It is also rich in vitamins E and K, which effectively fight skin problems and signs of aging and are often used against acne, wrinkles and various age spots. In this regard, the cultivation and demand for this oilseed is increasing every year [15]. Due to advances in cosmetology and biochemistry, rapeseed acts as a moisturizing and regenerating agent for the skin surface. To date, an innovative mixture of lipids has been obtained from rapeseed, contributing to the persistent softening of the stratum corneum of the epidermis. Rapeseed extract is used in particular in the production of sunscreen, moisturizing and anti-aging cosmetic products. Rapeseed contains large amounts of tocopherols, which provide intense protection against ultraviolet (UV) rays and help fight free radicals. Rapeseed-based sunscreens provide effective skin protection against harmful UV rays, as well as it has regenerating, moisturizing and rejuvenating properties [16]. The enzymatic technology used in the production of rapeseed oil results in the release of bioactive compounds suitable for skin care. Hydrolysates derived from rapeseed showed improved biological activity suitable for anti-aging skin formulations, namely antioxidant activity inhibiting approximately 80% of cellular reactive oxidative species, and anti-inflammatory and anti-wrinkle properties inhibiting approximately 36% of myeloperoxidase activity and more than 83% of elastase activity [17]. 

In Iranian traditional medicine, the roots of rapeseed have been used therapeutically as a diuretic agent in the treatment of scurvy and inflammation of the bladder. In addition, rapeseed is used in the treatment of liver and kidney diseases [18]. *Brassica* vegetable and oilseed consumption has been found to reduce age-related chronic diseases such as diabetes, kidney failure, hypertension and atherosclerosis. In addition, it contains various polyphenolic antioxidants, namely anthocyanin, kaempferol and quercetin, which are considered as the main components. Its other biologically active components include carotenoids, vitamins C and E, folic acid, sinapic acid, lignans, glucose esters, phenolic acid and tripeptides. Sinapic acid is an effective peroxynitrite scavenger that inhibits the modulation of necrosis or apoptosis pathways [19]. The antioxidant value of rapeseed depends on the genotype and different parts of the plant, environment and cooking methods. *Brassica* promotes several biological actions such as antiviral and anticarcinogenic effects by repairing oxidative DNA damage, inhibiting angiotensin converting enzyme activity, lowering serum low-density lipoprotein (LDL) levels and increasing glucose tolerance [20].

The medicinal properties of *Brassica napus* L. are poorly studied. Therefore, we studied the prospects of its use in pharmacy and medicine as a source of biologically active substances. This review differs from other articles because it considers different technological methods for obtaining extracts from *Brassica napus* L., the chemical composition of extracts, isolation methods of biologically active substances and pharmacological properties. Commonly used rapeseed extraction methods are supercritical, subcritical carbon dioxide extraction, conventional solid–liquid extraction, ultrasonic extraction and Soxhlet extraction methods. In this review, we offer a brief overview of the isolation methods of biologically active substances from rapeseed and the therapeutic potential of rapeseed in the treatment of various types of diseases. In many articles, the pharmacological properties of the individual components of rapeseed have been studied separately. Therefore, we conducted a review of all the biologically active components of *Brassica napus* L. and their therapeutic activity. In order to study the extraction and isolation methods of biocompounds from rapeseed and its pharmacological properties, we reviewed the literature using scientific search engines such as Scopus, Google Scholar, MDPI, Wiley Online and PubMed published between the years 1983 and 2022. The employed keywords were “*Brassica napus* L.”, “Rapeseed”, “Rapeseed oil”, “*Brassica napus* L. extract”, “*Brassica napus* L. seeds”, “Canola oil”, and/or “extraction methods”, “isolation”, “chemical composition”, “pharmacological activity”, “biocompounds” and “use in medicine”. We carried out a bibliographic search, considering articles, reviews, systematic reviews, books, clinical and preclinical studies on the use of rapeseed as a source of biologically active substances, as well as its use in medicine, the agro-industry and the food industry. Most publications about rapeseed were found on the subjects agriculture, biology and life sciences, chemistry and materials science, environmental and earth sciences, medicine and pharmacology, and food sciences. 

## 2. Technology for Obtaining Extracts from Rapeseed (*Brassica napus* L.)

### 2.1. Conventional Extraction Methods

Extraction is the first step to separate biologically active compounds from natural products. Extraction methods include solvent extraction, distillation method, pressing and sublimation according to the principle of extraction. Solvent extraction is the most widely used method. The extraction of natural products goes through the following stages: the solvent penetrates the solid matrix; the solute dissolves in solvents; the solute diffuses out of the solid matrix; the extracted solutions are collected. Any factor that increases diffusivity and solubility in the above steps will facilitate extraction. Extraction solvent properties, raw material particle size, solvent-to-solid ratio, extraction temperature and extraction duration will affect the extraction efficiency [21]. Rapeseed is used to obtain extracts by various extraction methods using solvents. The most commonly used solvent is hexane.

In one study using the soaking rapeseed extraction method, the various parts of the rapeseed were dried and ground into fine powders. Then, the solvent methanol 80% was added, mixed and left for 48 h. The extract obtained was filtered and the remaining solvent was evaporated. In this study, the antimicrobial activity of rapeseed extract was also studied, as a result of which the alcohol extract of rapeseed was active against *Pseudomonas aeruginosa* [22]. 

Aleksandra Szydłowska et al. obtained extracts from winter and spring rapeseed by ultrasonic and conventional solid–liquid extractions in order to study their antioxidant activity. In the conventional solid–liquid extraction process, 2 g of crushed seeds and 20 mL of methanol–water mixture (1:1 by volume) were transferred into a round bottom flask and shaken at room temperature for 30 min. The extraction was repeated three times and the residue was centrifuged. The extracts were filtered and stored in a refrigerator at 4 °C. In ultrasonic extraction, 2 g of crushed *Brassica napus* L. seeds and 15 mL of a 1:1 methanol–water mixture were placed in an ultrasonic bath with a frequency of 40 kHz, an ultrasonic input power of 180 W and a heating power of 800 W. Extraction was also carried out three times. The extract was filtered and the residue was centrifuged [23].

In the liquid–liquid extraction method, dried rapeseed leaf powder (400 g) was extracted by soaking at room temperature in 60% ethanol (*v*/*v*). The resulting extract was filtered and evaporated in vacuo at 40 °C using a rotary evaporator, obtaining an ethanolic extract (65.8 g) as a dark green residue. A part of the extract (30 g) was dissolved in the smallest amount of ethanol (~10 mL), suspended in distilled water and subjected to successive liquid–liquid extraction with petroleum ether, methylene chloride, ethyl acetate and n-butanol. The solvent ethanol was used in the extraction process. A one-factor experiment showed that 60% ethanol is suitable for extracting phenolic components from the plant. According to phytochemical screening, the leaves of *B. napus* L. contain flavonoids as the main component. The four flavonoid compounds were isolated as crystals by silica gel column chromatography and purified on Sephadex LH-20. Their types were established as quercetin, kaempferol, kaempferol-3-O-glucoside and quercetin-7-O-glucoside using UV and 1H-NMR methods [24].

In order to obtain extract via Soxhlet extraction, 5 g of rapeseed samples was dissolved in either 100 mL of water or 80% organic solution. Methanol, ethanol, acetone, butanol, chloroform and hexane were used as organic solvents. These solvents were mixed with the samples using a vortex and then continuously mixed with a slurry mixer for 12 h at room temperature, then centrifuged at 3000 rpm for 30 min. Supernatant samples were filtered using a syringe filter to maximize the recovery of the soluble extract. Lyophilization was carried out for 72 h, after which the samples were stored at –20 °C until required. This lyophilized material is the “extract” and is often used in all assays and experiments. All extracts were named according to the solvent used for food extraction, such as aqueous extract (WE), methanol extract (ME), ethanol extract (EE), acetone extract (AE), butanol extract (BE), chloroform extract (CE) and hexane extract (HE). All lyophilized extracts were mixed with 50% methanol and filtered to measure total phenols using the Folin–Ciocalteu reagent. All results were repeated three times. Meanwhile, water–methanol extracts showed higher quality recovery. In addition, extraction of rapeseed using only water without organic solvents increased the hydrolysis of sinapic acid conjugates (such as sinapin) and recovered higher amounts of free sinapic acid. HPLC-DAD-ABTS and LC-MS analyses revealed 47 compounds, identifying 32 compounds and 15 remained unknown. Aqueous extracts have proven to be the best for restoring the potentially health-beneficial glucosinolates. These results highlight the potential of rapeseed as a source of recovery of valuable phytochemicals for food and pharmaceutical purposes [25].

The best Soxhlet extraction efficiency was achieved using 60% methanol, 200 °C and a residence time of 20 min, which is >300%. The maximum yield of canolol was obtained using 100% methanol, 200 °C and 5 min with the addition of 1% NaOH in methanol (*w*/*v*). This study demonstrates that PSE (pressure extraction) can be an effective approach to both extract and transform phenolic compounds in rapeseed meal, which can be widely used in the extraction and transformation of natural bioactive compounds [26].

### 2.2. Modern and Greener Extraction Methods

Supercritical extraction was carried out in a CC-SFE device in which rapeseed and carbon dioxide (CO_2_) samples were preheated. CO_2_ was pumped through the bottom into the extraction column in a continuous stream for an extraction time of 60 min. The liquid sample was preliminarily placed inside the column. Extraction was carried out at 40 °C and 35 MPa. A chromatographic analysis of sterols by GC-MS was carried out, as a result of which the contents of β-sitosterol (50 wt.%, basic), campesterol (36.25 wt.%) and brassicasterol (23.91 wt.%) were determined [27].

Extraction of rapeseed was carried out using deionized water as a solvent at a ratio of solid and liquid phases of 1:20 g/mL. Samples were shaken in 500 mL Schott flasks in a water bath at 50 °C and 200 rpm for 30 min. For the hydrolysis of synapic acid esters (mainly sinapine) to free synapic acid chemical and enzymatic hydrolysis was used during the extraction of rapeseed meal. As a result, the following biologically active substances were found: synapic acid (9.8 ± 1.2 mg g^−1^), phytic acid (32.4 ± 1.8 mg g^−1^), protein (393.2 ± 18.6 mg g^−1^) and biomass (289.8 ± 16.2 mg g^−1^) [28].

Another study examined modified extraction methods using supercritical carbon dioxide extraction to produce high-value-added compounds from rapeseed oil deodorizing distillate and compared with modified Soxhlet extraction (solvent extraction + silica). For supercritical fluid extraction (SFE), the optimal extraction parameters were temperature 40 °C, pressure 350 bar (for phytosterols) and 400 bar (for tocopherol), 5% ethanol as co-solvent and pre-treatment with saponification. The optimized SFE procedure resulted in the recovery of three major phytosterols (50 wt.% β-sitosterol, 23.91 wt.% brassicasterol and 36.25 wt.% campesterol) and only α-tocopherol. In addition, comparative data showed that the extraction efficiency of phytosterols and tocopherols was about three times higher when using supercritical extraction compared to modified Soxhlet extraction. In addition, the use of ethanol as a co-solvent increased the recovery efficiency and purity of phytosterols and tocopherols [29].

The extraction of brassinosteroids was carried out by ultrasonic extraction, in which fresh plant tissues of rapeseed, frozen in liquid nitrogen, were crushed in a mortar with a pestle. Then, 50 mg of homogenized plant tissue was weighed into 2 mL Eppendorf tubes, and 1 mL of 60% (*v*/*v*) ACN, 2/3 of zirconium beads and an appropriate amount of stable isotope-labeled internal standards were added. The samples were homogenized with a ball mill at a frequency of 30 Hz for 3 min. Tubes with crude extract were sonicated for 5 min and extracted overnight with a laboratory rotator at 17 rpm and 4 °C [30].

Cvjetko et al. conducted the extraction process in a high-pressure laboratory extractor. First, the crushed sample weighing 30 g was placed into the extractor. The extracts were collected in pre-weighed glass vials and placed in a separator at room temperature and pressure. The studied pressure values varied from 20 MPa to 30 MPa and temperatures ranged from 40 °C to 60 °C at an extraction time of up to 4 h. No significant differences were found in the fatty acid profiles of rapeseed oil extracted with n-hexane Soxhlet and supercritical carbon dioxide extraction. n-Hexane and CO_2_ are non-polar solvents, so they show similar behavior when extracting chemical compounds from plant materials. The content of total unsaturated fatty acids in the studied rapeseed oil was very high, above 90%; the same can be said about the total content of monounsaturated fatty acids (MUFA), as it was above 65%. With the exception of palmitic acid, present at about 4.7%, and stearic acid, present at about 1.8%, the amount of other saturated fatty acids was much lower [31].

Uquiche et al. conducted supercritical SC-CO_2_ extraction on SPE-ed SFE by loading 27.1 g ground rapeseed meal (24.7 g dry substrate) into a 50 cm^3^ extraction vessel. The extraction pressure (20, 30 or 40 MPa) was adjusted manually using a pneumatically driven booster pump. The temperature of the air bath (oven) containing the extraction vessel (40, 50 or 60 °C) was in turn controlled automatically. The extract in the CO_2_ stream leaving the extraction vessel was separated from the solution using an expansion valve maintained at 110 °C and collected in pre-weighed glass vials (60 cm^3^ capacity). The resulting extract contains chemical compounds such as tocopherol, carotenoids, fatty acids (C14:0–C24:0) and sterols (brassicasterol, campesterol, β-sitosterol, etc.) [32].

For rapeseed cake, subcritical fluid extraction (SFE) is the best choice to maintain oil quality [33]. Several solvents such as propane, butane, dimethyl ether (DEM) and 1,1,1,2-tetrafluoroethane (R134a) can be used as subcritical fluids for the extraction of vegetable oil or animal lipids. Tingting Guo et al. developed and optimized the process of subcritical fluid extraction of rapeseed cake. In the experiment, the rapeseed cake was placed in an extraction vessel and covered with a lid. First, a vacuum pump was turned on to reduce the pressure in the extraction, separating and measuring vessels to −0.01 MPa. Then, the static extraction started. The R134a/butane was compressed with a compressor and the vacuum pump was opened for further reduction until the pressure in the three vessels reached −0.01 MPa. The extract was collected from the separator vessel and centrifuged. As a result, the optimal extraction parameters were identified: the ratio of butane R134a 1.5 kg/kg, extraction temperature 45 °C and extraction time 50 min. Thus, R134a/butane subcritical extraction is an effective method for extracting rapeseed cake and can potentially become an alternative to supercritical dioxide extraction and traditional hexane extraction [34].

As a result of studying various technologies for obtaining extracts from rapeseed, various types of biologically active compounds were discovered. The list of chemical compounds obtained by different extraction technologies is given in Table 1.

Among all the mentioned extraction methods, hexane extraction has the advantages of high productivity and low cost. However, oil extraction contains a lot of residual solvent. In addition, the high-temperature extraction and desolvation process destroys heat-sensitive substances in the oil [35]. Supercritical carbon dioxide extraction is also widely used in the extraction of oils and fats, which has the following advantages: low cost, non-toxic and environmentally friendly. The density, viscosity and diffusivity of CO_2_ can be controlled by adjusting the temperature and pressure of SCO_2_, achieving the goal of increasing the extraction rate or selectivity of oil and fat [36]. However, the pressure of supercritical extraction is high and the equipment is expensive, which hinders the wide use of the technology in industry [37]. Subcritical fluid extraction (SFE) is a new separation technology developed after supercritical fluid extraction. This guarantees quality and productivity, and promotes industrialization. Therefore, for rapeseed meal, SFE is the best choice to maintain oil quality.

## 3. Biologically Active Compounds in Rapeseed (*Brassica napus* L.)

Rapeseed (*Brassica napus* L.) contains a large amount of biologically active substances with therapeutic and prophylactic properties. This plant contains brassinosteroids, tocopherols, carotenoids, flavonoids, glucosinolates, vitamin C, minerals and fatty acids. 

### 3.1. The Content of Sterols in Rapeseed

Sterols and sterol esters of fatty acids predominate among the non-acylglycerol lipids of vegetable oils. Total sterols (the sum of esterified and non-esterified sterols) typically make up 0.2–1.0% of total lipids for most vegetable oils. Phytosterol is a class of chemical compounds with cyclopentane and phenanthrene as the backbone which perform physiological functions such as reducing the incidence of heart disease, anticancer and immune regulation [38]. The biological properties of sterols, particularly the ability to lower blood cholesterol levels, have led to significant interest in these compounds in the pharmaceutical and food industries. They inhibit the absorption of dietary cholesterol in the colon, as they compete with this compound for space in the micelle emulsion that transports systemic lipids from the blood to the liver. This action has a beneficial effect on the entire body, as it helps to maintain the full activity and health of the heart, prostate gland, liver and immune system [39]. Rapeseed oil contains 0.5–1.1% phytosterols, of which the total phytosterol fractions consist of 45–60% sitosterol, 25–39% campesterol, 5–13% brassicasterol, 3–7% avenasterol and 1% stigmasterol [40]. The total content of phytosterols in rapeseed oil (4.6–9.0 mg/g) is approximately twice that of sunflower (2.1–4.5 mg/g) or soybean (2.3–4.7 mg/g) oils [41]. Marzena Gawrysiak et al. showed the results of determining the content of phytosterols in the composition of rapeseed. In *Brassica napus* L. seeds harvested from the field, the total content of phytosterols in the analyzed species was 10.97 g/kg of fat. The dominant sterols were β-sitosterol (5.19 g/kg), which accounted for 47% of the total sterol content, and campesterol (4.48 g/kg), which accounted for 41% of the sterol fraction. Brassicasterol (0.96 g/kg), a characteristic sterol of cruciferous plants, in oils extracted from seeds, accounted for 8.5% of all sterols. Other sterols are found in much smaller amounts. The content of stigmasterol was 0.07 g/kg, and avenasterol was 0.27 g/kg of oil, corresponding to sterol content of 1% and 2.5%, respectively. Thus, among all identified sterols, the highest degradation rate was observed for stigmasterol and brassicasterol [42]. 

**Figure 1:**
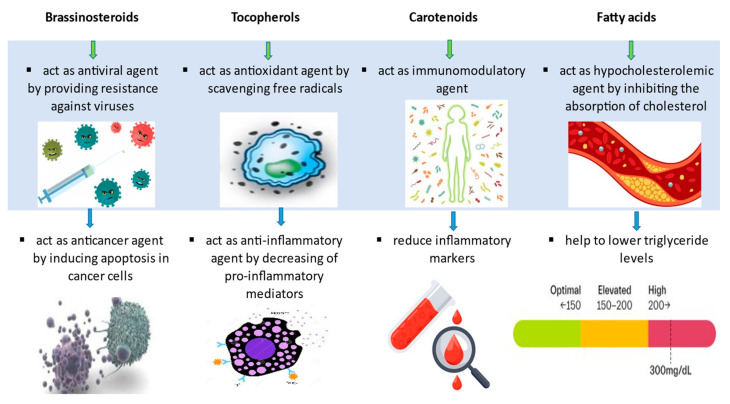
Pharmacological activity of rapeseed biocompounds [43,44,45,46].

Sterols, tocopherols and phenolic compounds found in rapeseed are biologically active components that exhibit an antioxidant effect. Figure 1 shows the composition of rapeseed and its mechanism of action. 

It is known that brassinosteroids have therapeutic properties against the development of cancer and have the potential for the development of new anticancer drugs [43]. These phytohormones also have antiproliferative, antiangiogenic, antiviral, antigenotoxic, antifungal and antibacterial effects on the human body [47]. The main aspect of the protective role of brassinosteroids is associated with their ability to provide resistance against viruses, especially herpes [48].

Some stigmasterol derivatives have been found to have a broad spectrum of antiviral activity in in vitro studies. These synthetic compounds have therapeutic properties, including the (i) inhibition of RNA virus replication and (ii) containment of HSV-1 infections [49]. Similar synthetic brassinosteroids, including stigmastane and androstane derivatives, are widely known immunomodulatory agents that also act as anti-inflammatory steroids [50].

In addition, rapeseed contains several biologically active compounds with important health benefits, including tocopherols, flavonoids, carotenoids and glucosinolates. Tocopherols exhibit the properties of vitamin E, are considered natural antioxidants and play an important role in improving human health. The content of tocopherols in rapeseed oils ranges from 45 to 75 mg %. High concentrations of α- and γ-tocopherol exist in refined rapeseed oil with total concentrations of tocopherols [51]. Vitamin E deficiency in the human body can lead to anemia, impaired immune response, retinopathy, neuromuscular and neurological problems [44]. Tocopherols have some potential health benefits, especially in the prevention of atherosclerosis, cancer, diabetes and obesity. In addition, these substances help reduce the risk of neurological and inflammatory diseases. Some studies show that α-tocopherol can reduce the risk of Parkinson’s disease, as well as have a preventive effect on Alzheimer’s disease [52].

### 3.2. The Content of Phenolic Compounds in Rapeseed

The main phenolic compound in rapeseed is sinapinic acid, which accounts for 70% of the total content of free phenolic acids and their derivatives such as sinapin [53]. Synapic acid also exists as glucopyranosyl synapate [54]. Only a small portion of sinapic acid, less than 16%, is present as free sinapic acid [55]. 2,6-dimethoxy-4-vinylphenol (vinylsyringol), known as canolol, is also one of the main phenols in rapeseed. It can be formed by decarboxylation of sinapinic acid and makes up 70–85% of the total amount of free phenolic acid [56]. Canolol has been shown to exhibit effective antioxidant and antimutagenic properties in the body. When *Brassica napus* L. seeds are pressed, only a small part of the phenols passes into crude oil, and most of them remain in the cake. Therefore, rapeseed cake is an excellent source of canolol [57].

Yue Wang et al. provided an analysis of the total content of phenols and flavonoids in a comparison of yellow- and black-seeded *B. napus* L. In this study, the content of phenolic compounds was significantly higher 5 weeks after flowering in black seeds (6.44 ± 0.97 mg EE/g phenols and 3.78 ± 0.05 mg EE/g flavonoids) than in yellow seeds (2.80 ± 0.13 mg/g phenols and 0.83 ± 0.01 mg/g flavonoids). HPLC analysis revealed 56 kinds of phenolic compounds, specifically kaempferol-3-O-glucoside, isorhamnetin-3-O-glucoside and quercetin-3-O-sophoroside, procyanidin B2 ([DP 2]), which were significantly lower in yellow seeds compared to the black seeds of *Brassica napus* L. In addition, an antioxidant capacity study was conducted in which iron reduction (FRAP) values were maximized at 5 WAF in black seeds (432.52 ± 69.98 µmol Fe(II)/g dry wt) and 6 WAF in yellow seeds (274.08 ± 2.40 µmol Fe(II)/g dry weight). As a result, antioxidant capacity was significantly reduced in yellow-seeded *B. napus* L. compared to black seeds, and a positive correlation between antioxidant and flavonoid content was found in both yellow- and black-seeded *B. napus* L. Thus, the black seeds of *B. napus* L. contain a large amount of phenolic compounds and have a pronounced antioxidant capacity compared to the yellow seeds of *B. napus* L. [58]. 

### 3.3. Vitamins and Glucosinolates in Rapeseed

The advantage of rapeseed oils is their rather high content of carotenoids, 0.30–0.57 mg %. Carotenoids are chemically related to vitamin A and are critical for skin and eye health, as well as for preventing oxidative-related disorders, including several types of cancer and cardiovascular disease [45]. β-carotene and lutein are the main carotenoids present in rapeseed oil. β-carotene has an antioxidant effect, and lutein helps prevent macular degeneration and is present in the eye to actively filter harmful light, reducing the amount of light reaching the retina [59]. Many carotenoids have anticarcinogenic, antimutagenic, antitoxic, immunomodulatory and other properties. This allows us to consider carotenoids as potential means of preventing the most common human diseases: atherosclerosis, malignant neoplasms and chronic infectious processes. Recently, special attention has been paid to carotenoids as promising agents for cancer chemoprevention. They help in inhibiting the growth of malignant tumors and inducing apoptosis. Additionally, they help lower blood pressure, reduce pro-inflammatory cytokines and reduce inflammatory markers [60].

Glucosinolates are sulfur- and nitrogen-containing secondary metabolites, some of which are well known for their anti-carcinogenic properties in humans [61]. Isothiocyanates (ITCs) are chemoprotectants produced as a product of the hydrolysis of glucosinolates by the enzyme myrosinase. ITCs present in cruciferous vegetables have higher anticancer properties and can inhibit cell proliferation [62]. ITCs inhibit cancer cell proliferation by inhibiting proteins involved in tumor initiation and proliferation pathways. Meanwhile, ITC treatment stimulates reactive oxygen species (ROS), cell cycle arrest, programmed cell death and autophagy [63]. Cruciferous consumption, both fresh and raw, appears to be more beneficial than cooked or boiled, as the bioavailability of ITC is higher in the former than in the latter. 

In addition, rapeseed is a rich source of vitamin C, minerals and fatty acids. Vitamin C performs important functions in the human body, such as ensuring the normal functioning of the immune system, the formation of red blood cells and collagen synthesis, and the absorption of iron from plant foods. In addition, ascorbic acid is an antioxidant; that is, it protects cells from damage by free radicals. A number of sources indicate that vitamin C is involved in more than 300 biological processes in the body. Vitamin C is not able to accumulate in the body, and any excess amount received from food or vitamin supplements is excreted in the urine and feces within a short period of time. Even the minimum amount of vitamin C is not created in the human body, it’s daily intake with food is necessary. The daily requirement for vitamin C of an adult is 50–100 mg [64].

### 3.4. Mineral Composition of Rapeseed

*Brassica napus* L. seeds contain a significant amount of mineral elements—Ca, P, K, Mg and Fe. In addition, they contain selenium, which has antioxidant properties and is necessary for the formation and metabolism of iodine-containing thyroid hormones [65]. Calcium is one of the main macroelements of the human body. Approximately 99% of calcium is found in bone tissue; the rest is found in extracellular fluid and other tissues. Sufficient calcium intake is important for the prevention and treatment of osteoporosis, as it helps to maintain bone mineral density, reduces the risk of hip fractures and potentiates the antiresorptive effect of estrogens on bone tissue [66]. Phosphorus is an essential mineral present in every cell of the human body. It is the second most abundant mineral after calcium, accounting for about 1% of total body weight. While the primary purpose of phosphorus is to build and maintain bones and teeth, it also plays an important role in the formation of DNA and RNA (the body’s genetic building blocks). This helps ensure that cells and tissues are properly maintained, repaired and replaced as they age. Potassium plays a role in the functioning of the nervous system, muscle contraction, maintaining the body’s water balance, maintaining normal blood pressure and blood sugar, and in many biochemical reactions that ensure human life. Normal levels of potassium in the body, in addition to stimulating regular heartbeats, can help offset the negative effects of a high-sodium diet, as potassium is actually an antagonist. This reduces the risk of developing hypertension, stroke and other cardiovascular diseases. Potassium helps maintain an alkaline environment in the body, which promotes bone health and muscle mass. Potassium may also help maintain normal kidney and adrenal function. A large number of studies demonstrate the health benefits of potassium, especially in relation to the cardiovascular system. The elemental composition of the human body consists of 99% of 12 basic chemical elements, among which magnesium ranks fourth after potassium, calcium and sodium. Being a necessary macroelement for cells and tissues, magnesium is involved in many physiological processes that ensure the normal functioning of the body: in the synthesis of enzymes (substrate of ATP, ADP, creatine kinase, hexokinase, etc.), direct activation of enzymes, regulation of cell membrane function (stabilization of cell membranes, cell adhesion, transmembrane flow of electrolytes), calcium antagonism (muscle contraction/relaxation, release of neurotransmitters, excitability of the specialized conduction system of the heart) and in plastic processes (protein synthesis and catabolism, exchange of nucleic acids and lipids, mitochondria) [67]. Iron is an essential trace element that is part of more than 100 enzymes in the human body and is involved in respiration, hematopoiesis, immunobiological processes and redox reactions. Its plasma concentration varies widely and in a healthy person is 10.8–28.8 µmol/L [68]. Iron is one of the main components of hemoglobin. Hemoglobin carries oxygen in the blood throughout the body. Iron is also involved in the process of reproduction of healthy red blood cells containing hemoglobin. Without iron, many processes in the body are impossible, including energy metabolism and DNA repair. Iron helps to keep the immune system in good shape, allowing the body to fight infection, and is involved in tissue growth. Iron deficiency can be due to the following reasons: chronic blood loss (hemorrhoids, profuse menorrhagia, etc.); insufficient intake of iron from food (vegetarianism); increased iron consumption (periods of intensive growth and development, pregnancy and lactation); malabsorption in pathology, the gastrointestinal tract or excessive use of phosphates, oxalates, calcium or tannin; and competitive iron consumption (helminthic infestations), as well as hypovitaminosis [69].

### 3.5. Fatty Acid Composition of Rapeseed

*Brassica napus* L. seeds contain a high fatty acid composition, especially high concentrations of mono- and polyunsaturated fatty acids, low concentrations (6.5–8%) of saturated fatty acids and a sufficient ratio of omega-3:omega-6 fatty acids [70]. The physical, chemical and nutritional properties of rapeseed oil obtained from *Brassica napus* L. seeds depend mainly on its fatty acid composition, which consists of approximately 60% oleic acid (C18:1), 4% palmitic acid (16:0) and 2% stearic acid [46]. Table 2 presents data on the content of fatty acids in rapeseed oil. 

Oleic acid is a monounsaturated fatty acid that belongs to the group of omega-9 unsaturated fatty acids. Oleic acid is the main monounsaturated fatty acid consumed by humans [79]. It is synthesized in the body from saturated fatty acids and partly from carbohydrates. The physiological need is 10% of the daily caloric intake. The metabolism of oleic acid has a number of features: human cells oxidize oleic acid at a rate that exceeds all fatty acids, especially palmitic acid; has the ability to reduce the sensitivity of low-density lipoproteins to lipid peroxidation; participates in the exchange of tocopherol, contributing to its antioxidant effect; and accelerates the incorporation of fatty acids into cell membranes [80].

Linoleic acid is a monobasic carboxylic acid with two isolated double bonds, and belongs to the omega-6 unsaturated fatty acids. Linoleic acid is one of the so-called essential fatty acids necessary for normal life; these acids enter the human and animal organism with food, mainly in the form of complex lipids—triglycerides and phosphatides. Linoleic acid belongs to the class of omega-6-unsaturated fatty acids; therefore, the human body is able to synthesize from it the quadruple unsaturated fatty acid arachidonic acid belonging to the same class. Human cell membranes contain on average 10 times more linoleic acid than omega-3-unsaturated α-linolenic fatty acid, which proves the critical importance of linoleic acid and the entire class of omega-6-unsaturated fatty acids for the normal functioning of cell and subcellular membranes [73].

Stearic acid (C18:0) is also a saturated fatty acid, found in varying amounts in all oils and fats, including marine oils, and is the main component of hydrogenated fats. Its main function is to supply the body with energy. Stearic acid favorably affects the functioning of internal organs: it participates in the thermoregulation of the body and improves the condition of hair and skin [74].

Palmitic saturated fatty acid is long-chain and abundant in nature. Palmitic acid plays an important role in maintaining the normal function of cell membranes and also helps the body store energy to facilitate metabolic functions. Some of these functions include providing membranes with the necessary characteristics for cell division, biological reproduction, and intracellular membrane transport. It also helps create sphingolipids found in cell membranes that help protect the brain and nerve cells [75].

Myristic, like other saturated fatty acids, has some antimicrobial activity, especially against Gram-positive bacteria. Myristic acid is able to potentiate the antibacterial action of antibiotics in the intestine, which can significantly increase the effectiveness of the treatment of acute intestinal infections of bacterial and viral–bacterial etiology. Myristic acid is an immunological stimulant when interacting with bacterial or viral antigens, helping to increase the body’s immune response to the introduction of an intestinal pathogen. During the formation of memory in the brain cells of experimental rats, the concentration of saturated fatty acids, especially myristic acid, increases [76].

Arachidonic acid is an organic compound, an omega-6 unsaturated fatty acid. The human body can independently synthesize it from the essential omega-6 unsaturated linoleic acid. Arachidonic acid is part of the phospholipids of the cell membranes of platelets and endothelial cells. Free arachidonic acid is rapidly metabolized to prostaglandins and thromboxanes. There are two main metabolic pathways for arachidonic acid, cyclooxygenase and lipoxygenase. The cyclooxygenase pathway of metabolism leads to the formation of prostaglandins and thromboxane A2, while the lipoxygenase pathway leads to the formation of leukotrienes. Arachidonic acid enters the body partly with food (vegetable oils) and is partly synthesized by the body, which ensures its constant presence in the human body [81].

Eicosanoic acid is a saturated fatty acid found in some vegetable oils and in small amounts in human milk. This acid helps maintain normal blood pressure by relaxing arteries and blood vessels and lowering blood lipids. Eicosanoic acid also reduces blood clotting factors. This acid is an integral part of the biological cell membrane, giving it the fluidity and flexibility necessary for the functioning of all cells, especially the nervous system, skeletal muscles and the immune system [77].

Behenic acid is a saturated fatty acid that is derived from the seeds of the indigenous Amazon tree species, Pentaclethra macroloba [82]. As a fatty acid, behenic acid is important to help provide a protective barrier against the environment in order to maintain good skin quality. In skincare, behenic acid has lubricant, emollient and soothing properties, which help to restore the skin’s natural oils and improve overall levels of hydration [78]. 

Erucic acid is an unsaturated carboxylic acid with one double bond, meaning that it is a monounsaturated, monoenoic acid. Erucic acid is toxic to the heart muscle; therefore, in different countries, legislative restrictions have been introduced so that the content of erucic acid in the oil used in food does not exceed 2–5%. This stimulated the development of low-erucic rapeseed varieties, from which rapeseed oil with a low content of erucic acid is obtained. It belongs to the class of omega-9 fatty acids, which means that it is not indispensable for the human body [83].

## 4. Pharmacological Properties and Therapeutic Activity of Rapeseed (*Brassica napus* L.)

**Figure 2:**
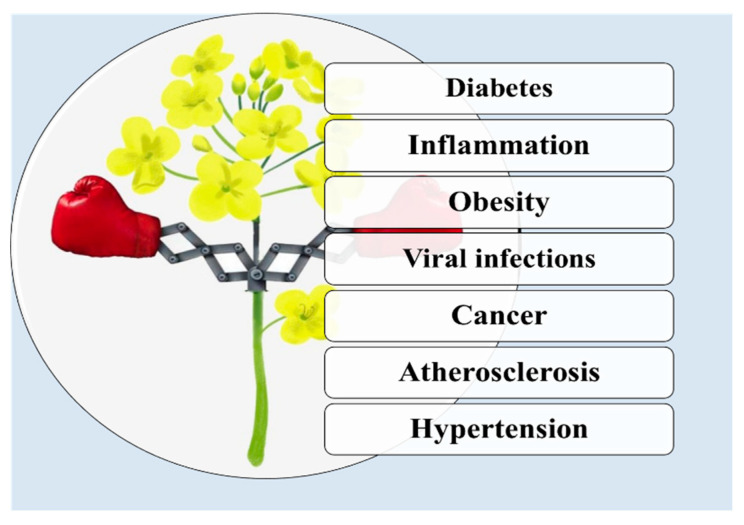
Therapeutic properties of rapeseed (*Brassica napus* L.) [84,85,86,87].

Rapeseed can be of great importance in the field of biomedicine, as it has the potential to prevent serious modern diseases, including diabetes, obesity and hypertension. Additionally, it is widely used in the treatment of kidney diseases, cystitis, abscesses, gout, joint pain and rheumatoid arthritis. Figure 2 presents data on the use of rapeseed in the treatment and therapy of various types of diseases.

### 4.1. Rapeseed in Diabetes and Obesity

Diabetes mellitus is one of the most common human diseases in the world, the frequency of which is increasing every year. This disease is associated with a violation of the absorption of glucose and develops due to a deficiency of the hormone insulin, resulting in the development of hyperglycemia, a persistent increase in blood glucose [88]. The disease is characterized by a chronic course, as well as a violation of all types of metabolism: carbohydrate, fat, protein, mineral and water–salt. Food products contain various types of carbohydrates. Some of them, such as glucose, consist of one six-membered heterocyclic carbohydrate ring and are absorbed in the intestine unchanged. These substances are broken down by various enzymes in the gastrointestinal tract into glucose molecules and other simple sugars, and are eventually also absorbed into the blood. Thus, glucose is the main carbohydrate of the blood and the whole organism. It plays an exceptional role in the metabolism of the human body: it is the main and universal source of energy for the whole organism. In case of insulin deficiency (type 1 diabetes mellitus) or a violation of the mechanism of interaction of insulin with body cells (type 2 diabetes mellitus), glucose accumulates in the blood in large quantities (hyperglycemia), and body cells (with the exception of insulin-independent organs) lose their main source of energy. To treat this disease, special enzymes are used that inhibit intestinal enzymes that break down complex carbohydrates to glucose, thereby reducing the absorption of glucose at the intestinal level. Currently, there are no conservative treatments that can cure type 2 diabetes mellitus [89]. In some studies, *Brassica napus* L. seeds have been found to contain angiotensin-converting enzyme (ACE) and dipeptidase-IV (DPP-IV) inhibitory peptides [90]. DPP-IV is a serine protease that helps inactivate glucagon-like peptide-1 when it enters the bloodstream. Preclinical studies show that these peptides exhibit glucose tolerance and high rates of insulin secretion. Thus, *Brassica napus* L. seeds have potential antidiabetic and antihypertensive properties [84].

Overweight and obesity are the main causes of diabetes and insulin resistance. Violation of the lipid profile is usually observed in patients with diabetes mellitus. Moreover, an increase in adipose tissue, especially visceral fat, is associated with the development of diabetes [91]. Due to the high content of flavonoids, the hypoglycemic properties of rapeseed have been studied. In one study, an experiment was carried out on Wistar rats, which were divided into five groups. Rapeseed extract was administered orally to rats for 4 weeks. As a result, rapeseed extract significantly lowered glucose levels in diabetic rats and helped lower serum triglyceride levels. Thus, rapeseed extract is beneficial for patients with diabetes and has a hypoglycemic effect [92].

The Westernization of eating habits in most countries is characterized by an increase in the consumption of high-calorie foods high in refined sugar, saturated fatty acids (SFAs) and an increased ratio of ω6/ω3 fatty acids. The associated increased prevalence of overweight, obesity and cardiovascular disease is a major public health problem. All these phenomena are the leading cause of disability and death. The consumption of refined oils as a source of lipids that contain high amounts of SFAs but do not contain other biologically active compounds increases the risk of developing these diseases. Dietary intake of cis-monounsaturated and polyunsaturated fatty acids and bioactive antioxidants such as vitamins and phenolic compounds is recognized as a cardioprotective and healthy metabolic effect [93]. Rapeseed is a good source of ω3 polyunsaturated fatty acids (PUFAs) for humans. The oil obtained from this plant contains 8–10% linolenic acid (ALA, 18:3ω3) and has a good ratio of ω6/ω3 acids [94]. *Brassica napus* L. seeds contain bioactive compounds, including antioxidant vitamins such as tocopherol (mainly alpha-tocopherol), phenolic molecules (canolol, sinapic acid, sinapin), coenzyme Q (CoQ) and phytosterols. These micronutrients have healthy metabolic, anti-inflammatory and physiological effects [95]. Consumption of fatty acids (especially PUFAs) increases the rate of fatty acid oxidation, resulting in peroxisomal and mitochondrial production of hydrogen peroxide. Antioxidant supplementation concomitantly with ω3 PUFAs is an appropriate nutritional strategy to reduce obesity-related metabolic disturbances by altering antioxidant activity and inflammation [96]. Frederic Capel et al. conducted preclinical trials to study the effect of rapeseed oil in the diet of rats in the fight against obesity. The diet consisted of palm oil and rapeseed oil enriched with polyunsaturated fatty acids and a sufficient ratio of ω6/ω3 acids. As a result, the rapeseed oil diet prevented glucose intolerance in rats and also reduced triacylglycerol levels. Thus, enriched rapeseed oil with natural micronutrients has been shown to improve skeletal muscle and adipose tissue metabolism, allowing better management of excess fatty acids and lowering glycemia, which may be beneficial in the long term [97]. 

Malgorzata Jamka et al. conducted a clinical trial to study the effect of amaranth and rapeseed oil on people suffering from overweight and obesity [98]. Exclusion criteria included a chronic systemic or gastrointestinal disease in anamnesis, liver disease, exocrine pancreatic insufficiency, drugs that affect fat digestion or absorption, pregnancy and lactation. All study participants were randomly assigned to groups I and II. In group I, amaranth oil was administered at a dose of 20 mL per day in the first intervention, and rapeseed oil at a dose of 20 mL per day was administered in the second intervention. Baseline variables included changes in tumor necrosis factor-alpha; adiponectin; oxidized low-density lipoprotein; apolipoproteins (Apo) A1, B and E; and markers of glucose and insulin homeostasis. Rapeseed oil had a greater positive effect on atherosclerosis markers than amaranth oil [99]. 

### 4.2. Rapeseed in Treatment of Cardiovascular Diseases

Cardiovascular disease is one of the main causes of premature death in many countries and leads to disability in patients. Among cardiovascular diseases, atherosclerosis is the most common pathological process. The progression of atherosclerosis is influenced by factors such as lipid abnormalities, oxidative stress and chronic inflammation [100]. Rapeseed oil is one of the main vegetable oils used in the food industry in many countries. This oil has a low amount of saturated fatty acids and a high amount of monounsaturated fatty acids compared to other edible oils. Additionally, rapeseed oil is a source of linoleic acid, α-linolenic acid and other essential fatty acids [101]. Various studies show that rapeseed oil can lower serum total cholesterol and low-density lipoprotein cholesterol. The phytosterols contained in rapeseed oil have a hypocholesterolemic effect by inhibiting the absorption of cholesterol [102]. Trace elements present in the composition of rapeseed oil can prevent the occurrence of atherosclerosis. One study conducted on laboratory rats examined the effect of rapeseed oil on risk factors for atherosclerosis. As a result of the study, after the consumption of oil in rats, the levels of triglycerides and plasma cholesterol decreased markedly. Thus, rapeseed oil prevents atherogenesis by improving plasma oxidative stress, the lipid profile and inflammation [103].

### 4.3. Antioxidant Properties of Rapeseed

Antioxidants are substances that have the ability to scavenge free radicals due to their redox properties. Natural antioxidants are safer and have fewer side effects compared to synthetic antioxidants [104]. Antioxidant activity, α-amylase inhibitory activity as well as antibacterial activity of rapeseed extract have been previously studied. When obtaining the extract, methanol was used as a solvent. The antioxidant activity of the extract was studied by DPPH analysis, in which at 125 (µg/m) extract, the DPPH value was 42.36 ± 3.26. The data obtained confirm the presence of antioxidant properties of the methanol extract of rapeseed. The high content of phenols and flavonoids in the rapeseed extract contributes to the manifestation of antioxidant properties [105]. Aleksandra Szydłowska-Czerniak et al. used three different analytical methods to determine the antioxidant capacity of rapeseed: ferric-reducing antioxidant power (FRAP), 2,2′-diphenyl-1-picrylhydrazyl (DPPH) and oxygen radical absorbance capacity (ORAC). Mean ORAC values for methanolic rapeseed extracts (4092–12,989 mmol Trolox/100 g) were significantly higher than FRAP and DPPH values (6218–7641 and 6238–7645 mmol Trolox/100 g, respectively) [106]. In another study, four modified methods were used to determine the antioxidant activity of rapeseed oil: 2,2-diphenyl-1-picrylhydrazyl (DPPH), 2,2′-azino-bis(3-ethylbenzothiazoline-6-sulfonic acid) (ABTS), cupric-reducing antioxidant capacity (CUPRAC) and ferric-reducing antioxidant power (FRAP). The obtained values of DPPH, ABTS, CUPRAC and FRAP of rapeseed oil were 126–586, 400–1998, 455–1913 and 72–291 µmol TE 100 g^−1^, respectively [107].

Azzurra Stefanucci et al. studied the antioxidant activity and enzymatic inhibitory ability of rapeseed extract. This extract contains flavonoids, glucosinolates, sinapic acid and disaccharides. According to the analysis of DPPH, the extract showed antioxidant activity as a result of studying the ability to scavenge radicals. The enzymatic inhibitory effect of the extract against acetylcholinesterase was studied using standard in vitro bioassays. In total, glucosinolates are healthy phytochemicals with antioxidant effects to the body [108].

Jovicic Dusica et al. studied the antioxidant activity of rapeseed leaves and roots at different stages of growth and grown under different field conditions. The study was conducted on the following parameters: the activity of the superoxide dismutase enzyme, the activity of glutathione peroxidase, the intensity of lipid peroxidation, the content of glutathione and the total antioxidant activity. As a result, the antioxidant activity of all studied parameters in all genotypes, both in leaves and roots, was higher in plants grown on Solonetz compared to plants grown on Chernozem [109].

### 4.4. Antiviral and Antibacterial Activity of Rapeseed

A subcritical water extract of rapeseed has been studied as an antiviral agent against the A/H1N1 virus. At maximum non-toxic concentrations, the subcritical aqueous extract showed antiviral activity against influenza A/H1N1 virus compared to other extracts of n-hexane, ethanol or hot water. The addition of 0.5 mg/mL subcritical aqueous extract to the culture medium resulted in 50.35% viability of kidney cells of dogs infected with influenza A/H1N1 virus. Thus, the extract had antiviral activity against influenza virus infection. The data obtained indicate the prospect of using rapeseed extract as an antiviral agent [110].

Hepatitis C virus is one of the main causes of chronic liver disease. With about 170 million cases in the world, it remains a serious public health problem [111]. Treatment of hepatitis C is ineffective in most cases, and is also long-term and expensive. At the same time, there is still no vaccine against this infection in practical healthcare. One study investigated the antiviral activity of an extract of a transgenic hepatitis C virus core protein derived from rapeseed and rHCVcp derived from *Escherichia coli*. Mice immunized with the transgenic core protein oil developed a strong humoral (IgG) and Th1-dependent cellular response, manifested by high levels of IFN-γ and a lower IgG1/IgG2a ratio and secretion of IL-4. The results of intracellular cytokine staining showed that immunization with transgenic core protein oil in mice triggered both CD4+ and CD8+ T cells to release IFN-γ, while CD4+ cells were mainly activated by Freund’s adjuvant. The data obtained are important for the development of a hepatitis C vaccine and indicate the potential of the antigen obtained from rapeseed [85,112].

Another study shows that the alcohol extract of rapeseed has an antibacterial effect on some types of pathogenic bacteria. The study was carried out using good diffusion agar and disk diffusion agar for *Staphylococcus aureus*, *Bacillus cereus*, *Escherichia coli* and *Pseudomonas aeruginosa*. A minimum inhibitory concentration and minimum bactericidal concentration test was performed using serial dilutions in vitro. As a result of this study, the alcohol extract of rapeseed prevented the growth of the above pathogenic bacteria, in which the minimum inhibitory concentration varied from 12.5 mg/mL to 100 mg/mL. Rapeseed oil has been used in the treatment of various types of diseases and skin infections in Pakistan. The oil is obtained from rapeseed by extraction with n-hexane. The antibacterial activity of rapeseed oil was studied on four microorganisms, namely *Staphylococcus aureus*, *Staphylococcus epidermidis*, *Pseudomonas aeruginosa* and *Klebsiella pneumonia*, causing some infections. In this study, rapeseed oil showed inhibitory activity against *Staphylococcus epidermidis*, *Pseudomonas aeruginosa* and *Klebsiella pneumonia*, but no inhibitory activity was found against *Staphylococcus aureus* [113].

Film with rapeseed extract obtained by supercritical carbon dioxide extraction also showed antibacterial activity against pathogenic microorganisms. An in vitro study was conducted in which the decimal reductive concentration and the minimum bactericidal concentration for pathogens were determined. Terpenoids, diterpenes and sesquiterpenes were found in the composition of this extract using gas chromatography. The study of antibacterial and fungicidal activity of rapeseed extract was carried out on four strains of pathogenic microorganisms, namely *S. aureus*, *E. coli*, *P. aeruginosa* and *C. albicans*. As a result, the rapeseed extract exhibited biological activity against *C. albicans* at a dilution of 1:32 and against *E. coli* at a dilution of 1:8. However, no inhibitory ability was found for *P. aeruginosa* and *S. aureus*. Thus, the data obtained indicate the potential of using rapeseed extract as an antibacterial agent in the pharmaceutical industry [114].

### 4.5. Anticancer Properties of Rapeseed

Cancer is considered one of the dysregulations of basic cellular functions such as growth signaling, anti-apoptotic signaling, gene stability and immune response [115]. Every year, the number of patients with cancer and the mortality rate from this disease increase [116]. Currently, *Brassica* plant metabolites are becoming new sources of anticancer therapy. Bioactive compounds in these plants exhibit anticancer activity against the main types of tumors [86]. Numerous clinical studies have been conducted to explore effective treatments for cancer. However, later, their toxic and side effects had a negative impact on patients. Radiation therapy and chemotherapy have serious side effects on healthy cells. Targeted therapies and immunotherapy are considered among the most effective cancer treatments, but they are applied to limited patients and are expensive therapies. In this regard, combination therapy replacing monotherapy has recently been used to prevent their side effects [117]. Moreover, searches are underway for new and effective drugs with reduced side and toxic effects. Plant metabolites are the source of a wide range of biological activities, such as anti-inflammatory, antimicrobial, anticancer and antianalgesic activity [118]. Among anticancer drugs, more than 60% of drugs are obtained from plant materials. Plants belonging to the *Brassicaceae* family are a rich source of glucosinolates, which are widely used as biologically active compounds [119]. Active metabolites of glucosinolates are used in various forms of cancer. For example, sulforaphane is used in the treatment of prostate cancer by helping to inhibit the growth of prostate cancer tumors. It also helps in the treatment of breast cancer, ovarian cancer and melanoma [120]. Erucin is effective in the treatment of pancreatic tumors, hepatocellular carcinoma and breast cancer. Indole-3-carbinol prevents colon cancer, hepatocellular carcinoma, breast cancer and prostate cancer [121].

It is now known that rapeseed may have chemoprotective and antitumor properties. Some researchers believe that the antitumor effect may be associated with the effective purification of reactive oxygen species by various rapeseed extracts [122]. In particular, brassinosteroids present in rapeseed extracts have an anticancer effect. The steroid fraction of the chloroform extract of rapeseed exhibits cytotoxic activity against certain types of human cancer cells. Cell death occurs due to the induction of caspase activity and inhibition of the Bcl-2 protein, mainly in prostate cancer cells [123]. Some results from other studies suggest that a diet based on omega-3 fatty acids helps to slow prostate tumorigenesis by lowering estradiol and testosterone levels by suppressing cell proliferation in C3(1) Tag mice [124]. Rape root extract has an anticancer effect by inhibiting the proliferation of human Hep G2 cancer cells. Other data indicate that γ-tocopherol in rapeseed extract may potentially reduce the risk of prostate cancer [125].

Breast cancer is one of the main oncological diseases frequently found in women in developed countries [126]. Some studies show that plant-derived protein hydrolysates can prevent cancer. Peptides are bioavailable chemical compounds compared to proteins and free amino acids. Thus, they can replace chemotherapy due to their effective tissue penetration and low toxicity [127]. Rapeseed meal is a by-product of the agro-industrial sector, which contains a large amount of proteins and peptides with anticancer properties. In one study, an enzymatic digest of rapeseed protein inhibited the proliferation of a breast cancer cell line. The protein isolate was extracted from the alkaline extract of rapeseed. As a result, all obtained protein hydrolysates exhibited an antiproliferative effect on MCF-7 cells [128].

### 4.6. Rapeseed in Treatment of Hypertension

Hypertension is high blood pressure, which is a serious pathological condition that significantly increases the risk of developing diseases of the cardiovascular system, brain, kidneys and other diseases. In the modern world, the prevalence of arterial hypertension is 30–45% among the adult population [129]. Blood pressure is the force exerted by circulating blood on the arteries, the most important blood vessels in the body. Hypertension is characterized by an excessive increase in blood pressure. It is predicted that the number of patients with this disease by 2025 will be 1.6 billion [130]. Rapeseed-derived peptides can be used to treat arterial hypertension. One in vivo study in mice examined the safety and antihypertensive properties of rapeseed peptides in synergy with captopril. According to toxicity studies, the maximum tolerated dose exceeded 25 g kg^−1^ body weight per day in mice, i.e., rapeseed peptides are non-toxic. Moreover, rapeseed peptides synergistically increased the amplitude of captopril lowering blood pressure by 9% and increased the time of antihypertensive activity in hypertensive rats by 20% [87]. Thus, rapeseed peptides can be used in the development of an antihypertensive agent that helps lower blood pressure.

Table 3 presents the main pharmacological properties and therapeutic value of rapeseed obtained from various studies. The pharmacological activity of some individual components characteristic of rapeseed has been studied by Hussain et al., Akbari et al., Batool et al. and Ferrero et al. Rapeseed has been studied as a source of biologically active substances that contribute to the prevention and treatment of various types of diseases.

Generally, rapeseed has pharmacological properties that contribute to the treatment of various forms of diseases such as diabetes, obesity, viral infections, bacterial diseases, atherosclerosis, cancer and hypertension. Phytochemical compounds present in rapeseed inhibit and reduce the proliferation of cancer cells, stimulate the immune system and have an antioxidant effect.

## 5. Conclusions and Future Perspectives

Rapeseed is a widespread oilseed crop that is used in food, agriculture, medicine and cosmetic industry. Extracts obtained from rapeseed contain a wide range of biologically active substances that can be used in the treatment of different types of diseases. The most commonly used extraction methods for obtaining extracts from *Brassica napus* L. seeds are supercritical and subcritical extraction processes. These extracts are characterized by a low level of saturated fatty acids and a significant amount of monounsaturated fatty acids and polyunsaturated fatty acids, which have a cardioprotective effect on the body and help to reduce blood cholesterol levels. Extracts also contain large amounts of phytosterols, carotenoids, tocopherols and flavonoids. These substances have a positive effect on the cardiovascular and nervous systems, prevent the formation of blood clots and effectively fight infectious diseases. Rapeseed is widely studied for medicinal purposes as an anti-inflammatory, antiviral, antidiabetic, anticancer and antioxidant agent. The data in this review illustrate the prospects of using rapeseed to obtain extracts with a wide range of biologically active substances and in the treatment of various types of diseases. 

## Figures and Tables

**Table 1:** Extracted biologically active compounds from rapeseed (*Brassica napus* L.).

Technologies for Obtaining Extracts from Rapeseed	Extraction Parameters	Methods for Determining Biologically Active Substances	Pharmacological Activity	Isolated Compounds	Reference
Soaking method	Solvent is methanol 80%, extraction time 48 h	-	The extract has antimicrobial activity against *Pseudomonas aeruginosa*	-	[22]
Ultrasonic extraction	Solvent is methanol–water, ultrasonic bath frequency 40 kHz, ultrasound power 180 W and heating power 800 W	-	The extract exhibits antioxidant activity	-	[23]
Conventional solid–liquid extraction	Solvent is methanol–water, at room temperature with an extraction time of 30 min	-	The extract has an antioxidant effect	-	[23]
Liquid-liquid extraction	Solvent is ethanol 60%, at room temperature, extract yield 65.8 g	Spectral methods, UV and 1H-NMR techniques	The extract contains phenolic compounds in its composition	Quercetin, kaempferol, kaempferol-3-O-glucoside, quercetin-7-O-glucoside	[24]
Soxhlet extraction	Solvents: methanol, ethanol, acetone, butanol, chloroform, hexane. Extraction time: 12 h at room temperature	High-performance liquid chromatography-diode array detection	Aqueous extracts contain large amounts of glucosinolates	Synapic acids	[25]
Pressurized solvent extraction (PSE)	Solvent is methanol 60%, extraction temperature 200 °C, extraction time 20 min	1H NMR	Methanol extract is most suitable for extracting phenolic compounds	Sinapine thiocyanate, sinapic acid, canolol	[26]
Supercritical CO_2_ extraction	Extraction time 60 min, extraction temperature 40 °C, pressure 35 MPa	High-performance liquid chromatography	The extract contains a large amount of sterols	β-sitosterol, campesterol, brassicasterol	[27]
Solid–liquid extraction	Solvent is deionized water, temperature 50 °C, extraction time 30 min	High-performance liquid chromatography	The extract contains a large amount of synapic acid esters	Synapic acid, phytic acid, protein	[28]
Supercritical CO_2_ extraction	Temperature 40 °C, pressure 350 bar (for phytosterol), 440 bar (for tocopherol), co-solvent: ethanol 5%	Gas chromatography	The extract contains a large amount of phytosterols and tocopherols	β-sitosterol, brassicasterol, campesterol, α-tocopherol	[29]
Supercritical CO_2_ extraction	Pressure 20–30 MPa, temperature 40–60 °C, extraction time 4 h	Gas chromatography	The extract contains a large amount of unsaturated fatty acids	Oleic acid, stearic acid, linoleic acid	[31]
Supercritical CO_2_ extraction	Pressure 20, 30 and 40 MPa; temperature 40, 50 and 60 °C	UV spectrophotometry, gas chromatography	The extract contains a large amount of fatty acids	Tocopherols, carotenoids, sterols, fatty acids	[32]
Subcritical fluid extraction (SFE)	The ratio of butane R134a 1.5 kg/kg, extraction temperature 45 °C and extraction time 50 min	Gas chromatography, high-performance liquid chromatography	The extract had the highest tocopherols and β-carotene, higher canolol and phytosterols but fewer phospholipids	Phospholipids, fatty acids, β-carotene, tocopherols, phytosterols	[34]

**Table 2:** Fatty acid composition of rapeseed oil.

Fatty Acids	Pharmacological Activity	Quantity, %	References
α-linolenic acid	Reduces the risk of cardiovascular disease	10.34 ± 0.91	[71]
Palmitoleic acid	Improves cognitive functions and has a positive effect on the brain	0.28 ± 0.01	[71]
Erucic acid	-	0.03 ± 0.01	[71]
Heptadecanic acid	Shows protective effect on the epidermis	0.05 ± 0.02	[72]
Gadoleic acid	Shows moisturizing effect on the skin	1.29 ± 0.18	[72]
Oleic acid	Shows antioxidant effect	55.22 ± 0.85	[72]
Linoleic acid	Involved in normal functioning of cell and subcellular membranes	24.24 ± 1.13	[73]
Stearic acid	Promotes the development of the nervous system and thermoregulation of the body	2.08 ± 0.09	[74]
Palmitic acid	Maintain the normal function of cell membranes	6.06 ± 0.18	[75]
Myristic acid	Shows antimicrobial activity	0.25 ± 0.01	[76]
Arachidic acid	Helps prevent the development of stomach ulcers	0.27 ± 0.01	[77]
Eicosaenoic acid	Helps to maintain normal blood pressure	1.00 ± 0.03	[77]
Behenic acid	Helps to improve overall levels of hydration in skin	0.23 ± 0.01	[78]

**Table 3:** Overview of pharmacological properties of rapeseed (*Brassica napus* L.).

Pharmacological Properties	Therapeutic Activity of Rapeseed	Concentration	Reference
Antidiabetic properties	The peptides present in rapeseed affect high levels of insulin secretion	680 µg/mL	[84]
Hypoglycemic action	An extract derived from rapeseed significantly reduces serum triglyceride levels	3 g/kg	[92]
Atherosclerosis	Rapeseed oil prevents atherosclerosis by lowering plasma triglyceride and cholesterol levels	20%	[103]
Antioxidant activity	The methanol extract of rapeseed has an antioxidant effect	1000 μL	[105]
Antiviral activity	An aqueous extract of rapeseed shows antiviral activity against the A/H1N1 influenza virus	0.5 mg/mL	[110]
Hepatitis C virus	The extract of transgenic core protein of hepatitis C virus obtained from rapeseed shows activity against hepatitis C virus	0.05%	[112]
Antibacterial properties	The alcohol extract of rapeseed has an antibacterial effect on some types of pathogenic bacteria	75 µL	[113]
Anticancer properties	Rapeseed root extract shows an anticancer effect by inhibiting the proliferation of human cancer cells Hep G2	3.75–10 mg/mL	[125]
Hypertension	Rapeseed-derived peptides shows antihypertensive effects	1.27 mg mL^−1^	[87]

## Data Availability

Not applicable.

## References

[1] Iniguez-Luy F.L., Federico M.L., Schmidt R., Bancroft I. (2011). The Genetics of *Brassica napus*. Genetics and Genomics of the Brassicaceae.

[2] Tetteh E.T., de Koff J.P., Pokharel B., Link R., Robbins C. (2019). Effect of Winter Canola Cultivar on Seed Yield, Oil, and Protein Content. Agron. J..

[3] Orazbayev K., Zheksembiev R.K., Digarbayeva A.M. (2011). Cultivation of rapeseed as an intermediate fodder crop in the foothill irrigated conditions of the Zailiysky Alatau. Vestn. KazNU. Biol. Ser..

[4] Yerzhanova S.T., Meirman G.T., Abayev S.S., Shegebayev G.O., Aynebekova B.A., Kaskabayev N.B. Winter rapeseed is a perspective crop in the Southern and South-Eastern regions of Kazakhstan. Proceedings of the “Actual Problems of Agro-Science in the Context of Adaptation to Global Climate Change” Devoted to the 75th Anniversary of the Doctor of Agricultural Sciences, Professor, Academician of National Academy of Sciences and Academy of Agricultural of Sciences of the Republic of Kazakhstan Meiirman Galiolla.

[5] Malik R. (1990). Prospects for Brassica carinata as an oilseed crop in India. Exp. Agric..

[6] Salunkhe D.K. (1992). World Oilseeds: Chemistry, Technology, and Utilization.

[7] Di Lena G., Sanchez del Pulgar J., Lucarini M., Durazzo A., Ondrejíčková P., Oancea F., Frincu R.-M., Aguzzi A., Ferrari Nicoli S., Casini I. (2021). Valorization Potentials of Rapeseed Meal in a Biorefinery Perspective: Focus on Nutritional and Bioactive Components. Molecules.

[8] Future Market Insights (2018) Rapeseed Oil Market: Industrial Applications of Rapeseed Oil in Biodiesel Production to Compete with Its Use in Food Processing: Global Industry Analysis (2013–2017) & Opportunity Assessment (2018–2027). https://www.futuremarketinsights.com/reports/rapeseed-oil-market.

[9] STATISTA Worldwide Oilseed Production since 2008. https://www.statista.com/statistics/267271/worldwideoilseed-production-since-2008.

[10] Arrutia F., Binner E., Williams P., Waldron K.W. (2020). Oilseeds beyond oil: Press cakes and meals supplying global protein requirements. Trends Food Sci. Technol..

[11] Yates K., Pohl F., Busch M., Mozer A., Watters L., Shiryaev A., Kong Thoo Lin P. (2019). Determination of sinapine in rapeseed pomace extract: Its antioxidant and acetylcholinesterase inhibition properties. Food Chem..

[12] El-Beltagi H.E.S., Mohamed A.A. (2010). Variations in fatty acid composition, glucosinolate profile and some phytochemical contents in selected oil seed rape (*Brassica napus* L.) cultivars. Fats Oil.

[13] Rabiej-Kozioł D., Tymczewska A., Szydłowska-Czerniak A. (2022). Changes in Quality of Cold-Pressed Rapeseed Oil with Sinapic Acid Ester-Gelatin Films during Storage. Foods.

[14] Raboanatahiry N., Li H., Yu L., Li M. (2021). Rapeseed (*Brassica napus*): Processing, Utilization, and Genetic Improvement. Agronomy.

[15] Thiyam-Holländer U., Eskin N.A.M., Matthäus B. (2012). Canola and Rapeseed: Production, Processing, Food Quality, and Nutrition.

[16] Tileuberdi N.N., Torgauytova N., Turgumbayeva A.A. (2020). Collection of Materials of the X All-Russian Scientific Conference of Students and Graduate Students with International Participation “Young Pharmacy–the Potential of the Future”.

[17] Rivera D., Rommi K., Fernandes M.M., Lantto R., Tzanov T. (2015). Biocompounds from rapeseed oil industry co-stream as active ingredients for skin care applications. Int. J. Cosmet. Sci..

[18] Saeidnia S., Gohari A.R. (2012). Importance of *Brassica napus* as a medicinal food plant. J. Med. Plants Res..

[19] Huang J.F., Zheng X.Q., Lin J.L., Zhang K., Tian H.J., Zhou W.X., Wang H., Gao Z., Jin H.M., Wu A.M. (2020). Sinapic Acid Inhibits IL-1β-Induced Apoptosis and Catabolism in Nucleus Pulposus Cells and Ameliorates Intervertebral Disk Degeneration. J. Inflamm. Res..

[20] Fernández S., Pilar M.D., Pérez M., Velasco Pazos T., Cartea González P., Elena M. (2011). Antioxi-dant properties of Brassica vegetables. Funct. Plant. Sci. Biotechnol..

[21] Zhang Q.W., Lin L.G., Ye W.C. (2018). Techniques for extraction and isolation of natural products: A comprehensive review. Chin. Med..

[22] Katayoon D., Akram T., Mahdi V. Investigation of Antipseudomanal Activity of *Brassica Napus* L.. Proceedings of the International Proceedings of Chemical, Biological & Environmental Engineering.

[23] Szydłowska-Czerniak A., Tułodziecka A. (2014). Antioxidant Capacity of Rapeseed Extracts Obtained by Conventional and Ultrasound-Assisted Extraction. J. Am. Oil Chem. Soc..

[24] Abaza A.M. (2018). Isolation and identification of defensive flavonoids from Brassica napus leaves extract with promising biological activity to control the cotton leafworm, Spodoptera littoralis (boisd.). Egypt. J. Agric. Res..

[25] Hussain S., Rehman A.U., Obied H.K., Luckett D.J., Blanchard C.L. (2022). Extraction, Chemical Characterization, In Vitro Antioxidant, and Antidiabetic Activity of Canola (*Brassica napus* L.) Meal. Separations.

[26] Li J., Guo Z. (2016). Concurrent extraction and transformation of bioactive phenolic compounds from rapeseed meal using pressurized solvent extraction system. J. Ind. Crops Prod..

[27] Asl P.J., Niazmand R., Jahani M. (2019). Theoretical and experimental assessment of supercritical CO_2_ in the extraction of phytosterols from rapeseed oil deodorizer distillates. J. Food Eng..

[28] Thiel A., Muffler K., Tippkötter N., Suck K., Sohling U., Hruschka S.M., Ulber R. (2015). A novel integrated downstream processing approach to recover sinapic acid, phytic acid and proteins from rapeseed meal. J. Chem. Technol. Biotechnol..

[29] Asl P.J., Niazmand R., Yahyavi F. (2020). Extraction of phytosterols and tocopherols from rapeseed oil waste by supercritical CO_2_ plus co-solvent: A comparison with conventional solvent extraction. Heliyon.

[30] Tarkowská D., Strnad M. (2017). Protocol for Extraction and Isolation of Brassinosteroids from Plant Tissues. Methods Mol. Biol..

[31] Cvjetko M., Jokić S., Lepojević Ž., Vidović S., Marić B., Radojčić Redovniković I. (2012). Optimization of the Supercritical CO_2_ Extraction of Oil from Rapeseed Using Response Surface Methodology. Food Technol. Biotechnol..

[32] Uquiche E., Romero V., Ortíz J., Del Valle J.M. (2012). Extraction of oil and minor lipids from cold-press rapeseed cake with Supercritical CO_2_. Braz. J. Chem. Eng..

[33] Russin T.A., Boye J.I., Arcand Y., Rajamohamed S.H. (2011). Alternative techniques for defatting soy: A practical review. Food Bioprocess. Technol..

[34] Guo T., Wan C., Huang F. (2019). Extraction of rapeseed cake oil using subcritical R134a/butane: Process optimization and quality evaluation. Food Sci. Nutr..

[35] Campbell K.A., Glatz C.E., Johnson L.A., Jung S., de Moura J.M.N., Kapchie V., Murphy P. (2011). Advances in aqueous extraction processing of soybeans. J. Am. Oil Chem. Soc..

[36] Jokić S., Bijuk M., Aladić K., Bilić M., Maja M. (2016). Optimization of supercritical CO_2_ extraction of grape seed oil using response surface methodology. Int. J. Food Sci. Technol..

[37] Sahena F., Zaidul I., Jinap S., Karim A.A., Abbas K.A., Norulaini N., Omar A. (2009). Application of supercritical CO_2_ in lipid extraction—A review. J. Food Eng..

[38] Yang M., Zheng C., Zhou Q.I., Liu C., Li W., Huang F. (2014). Influence of microwaves treatment of rapeseed on phenolic compounds and canolol content. J. Agric. Food Chem..

[39] Normén L., Frohlich J., Trautwein E., Dutta P.C. (2003). Role of plant sterols in cholesterol lowering. Phytosterols as Functional Food Components and Nutraceuticals.

[40] Hamama A.A., Bhardwaj H.L., Starner D.E. (2003). Genotype and growing location effects on phytosterols in canola oil. J. Am. Oil Chem. Soc..

[41] Vlahakis C., Hazebroek J. (2000). Phytosterol accumulation in canola, sunflower, and soybean oils: Effects of genetics, planting location, and temperature. J. Am. Oil Chem. Soc..

[42] Gawrysiak-Witulska M., Rudzińska M., Wawrzyniak J., Siger A. (2012). The Effect of Temperature and Moisture Content of Stored Rapeseed on the Phytosterol Degradation Rate. J. Am. Oil Chem. Soc..

[43] Malíkova J., Swaczynova J., Kolar Z., Strnad M. (2008). Anticancer and antiproliferative activity of natural brassinosteroids. Phytochemistry.

[44] Shahidi F., de Camargo A.C. (2016). Tocopherols and Tocotrienols in Common and Emerging Dietary Sources: Occurrence, Applications, and Health Benefits. Int. J. Mol. Sci..

[45] Fernández-García E., Carvajal-Lérida I., Jarén-Galán M., Garrido-Fernández J., Pérez-Gálvez A., Hornero-Méndez D. (2012). Carotenoids bioavailability from foods: From plant pigments to efficient biological activities. Food Res. Int..

[46] Bauer B., Kostik V., Gjorgjeska B. (2015). Fatty acid composition of seed oil obtained from different canola varieties. Farm. Glas..

[47] Michelini F.M., Zorrilla P., Robello C., Alche L.E. (2013). Immunomodulatory activity of an anti-HSV-1 synthetic stigmastaneanalog. Bioorganic Med. Chem..

[48] Calil I.P., Fontes E.P. (2016). Plant immunity against viruses: Antiviral immune receptors in focus. Ann. Bot..

[49] Romanutti C., Castilla V., Coto C.E., Wachsman M.B. (2007). Antiviral effect of a synthetic brassinosteroid on the replication of vesicular stomatitis virus in Vero cells. Int. J. Antimicrob. Agents.

[50] Kaur Kohli S., Bhardwaj A., Bhardwaj V., Sharma A., Kalia N., Landi M., Bhardwaj R. (2020). Therapeutic Potential of Brassinosteroids in Biomedical and Clinical Research. Biomolecules.

[51] Li C., Yao Y., Zhao G., Cheng W., Liu H., Liu C., Shi Z., Chen Y., Wang S. (2011). Comparison and analysis of fatty acids, sterols, and tocopherols in eight vegetable oils. J. Agric. Food Chem..

[52] Li F.J., Shen L., Ji H.F. (2012). Dietary intakes of vitamin E, vitamin C, and beta-carotene and risk of Alzheimer’s disease: A meta-analysis. J. Alzheimer’s Dis..

[53] Kozlowska H., Zadernowski R., Sosulski F. (1983). Phenolic acid in oilseed flours. Nahrung/Food.

[54] Amarowicz R., Shahidi F. (1994). Chromatographic separation ofglucopyranosyl sinapate from canola meal. JAOCS J. Am. Oil Chem. Soc..

[55] Kozlowska H., Naczk M., Shahidi F., Zadernowski R., Shahidi F. (1990). Phenolic Acids and Tannins in Rapeseed and Canola. Canola and Rapeseed.

[56] Wakamatsu D., Morimura S., Sawa T., Sawa T., Kida K., Nakai C., Maeda H. (2005). Isolation, identification, and structure of a po-tent alkyl-peroxyl radical scavenger in crude canola oil, canolol. Biosci. Biotechnol. Biochem..

[57] Kuwahara H., Kanazawa A., Wakamatu D., Morimura S., Kida K., Maeda H. (2004). Antioxidative and Antimutagenic activities of 4-Vinyl-2, 6-dimethoxyphenol (Canolol) Isolated from Canola Oil. J. Agric. Food Chem..

[58] Wang Y., Meng G., Chen S., Chen Y., Jiang J., Wang Y.-P. (2018). Correlation Analysis of Phenolic Contents and Antioxidation in Yellow- and Black-Seeded Brassica napus. Molecules.

[59] Granado F., Olmedilla B., Blanco I. (2003). Nutritional and clinical relevance of lutein in human health. Br. J. Nutr..

[60] Sergeyev A.V., Ananev V.S., Kapitanov A.B., Korostylev S.A., Bukreev Y.M., Vlasenkova N.K., Prosalkova I.R., Reshetnikova V.V., Shubina I.Z. (2017). Pharmacokinetics of carotenoids and carotene-containing drugs. Russ. J. Biother..

[61] Soundararajan P., Kim J.S. (2018). Anti-Carcinogenic Glucosinolates in Cruciferous Vegetables and Their Antagonistic Effects on Prevention of Cancers. Molecules.

[62] Higdon J.V., Delage B., Williams D.E., Dashwood R.H. (2007). Cruciferous vegetables and human cancer risk: Epidemiologic evidence and mechanistic basis. Pharmacol. Res..

[63] Hecht S.S. (2000). Inhibition of carcinogenesis by isothiocyanates. Drug Metab. Rev..

[64] Katalova E.A., Penzina T.N. (2018). Sources of Vitamin C. Scientist.

[65] Mhitaryanc L.A., Mhitaryanc G.A., Marasheva A.N., Timofeenko T.I. (2012). Features of the chemical composition of rape seeds of modern breeding varieties. News of higher educational institutions. Food Technol..

[66] Lutsenko A.S., Rozhinskaya L.Y., Toroptsova N.V., Belaya Z.E. (2017). The role of calcium and vitamin D medications in prevention and treatment of osteoporosis. Osteoporos. Bone Dis..

[67] Trisvetova E.L. (2012). Magnesium in clinical practice. Ration. Pharmacother. Cardiol..

[68] Cadet E., Gadenne M., Capront D., Rochette J. (2005). Donnes recentes sur metabolisme du fer: Un etat de transition. Rev. Med. Interne.

[69] Vatutin N.T., Kalinkina N.V., Smirnova A.S., Kashanskaya O.K., Milner I.A. (2012). The role of iron in the human body. Bulletin of V.N. Karazin Kharkiv National University. Ser. Med..

[70] Flakelar C.L., Adjonu R., Doran G., Howitt J.A., Luckett D.J., Prenzler P.D. (2022). Phytosterol, Tocopherol and Carotenoid Retention during Commercial Processing of Brassica napus (Canola) Oil. Processes.

[71] Sagan A., Blicharz-Kania A., Szmigielski M., Andrejko D., Sobczak P., Zawiślak K., Starek A. (2019). Assessment of the Properties of Rapeseed Oil Enriched with Oils Characterized by High Content of α-linolenic Acid. Sustainability.

[72] Pospišil M., Škevin D., Mustapić Z., Neđeral Nakić S., Butorac J., Matijević D. (2007). Fatty Acid Composition in Oil of Recent Rapeseed Hybrids and 00 Cultivars. Agric. Conspec. Sci..

[73] Severin E.S. (2003). Biochemistry.

[74] Volovic V.T., Leonidova T.V., Korovina L.M., Blohina N.A., Kasarina N.P. (2019). Comparison of the fatty acid composition of various edible oils. Int. J. Appl. Basic Res..

[75] Titov V.N., Dygai A.M., Kotlovskiy M.Y., Kurdoyak Y.V., Yakimenko A.V., Yakimovich I.Y., Aksyutina N.V., Kotlovskiy Y.V. (2014). Palmitic and oleic acids and their role in pathogenesis of atherosclerosis. Bull. Sib. Med..

[76] Wallis T.P., Venkatesh B.G., Narayana V.K., Kvaskoff D., Ho A., Sullivan R.K., Windels F., Sah P., Meunier F.A. (2021). Saturated free fatty acids and association with memory formation. Nat. Commun..

[77] Tokuda H., Kontani M., Kawashima H., Kiso Y., Shibata H., Osumi N. (2014). Differential effect of arachidonic acid and docosahexaenoic acid on age-related decreases in hippocampal neurogenesis. Neurosci. Res..

[78] Raghallaigh S.N., Bender K., Lacey N., Brennan L., Powell F. (2012). The fatty acid profile of the skin surface lipid layer in papulopustular rosacea. Br. J. Dermatol..

[79] Gardner A.S., Rahman I.A., Lai C.T., Hepworth A., Trengove N., Hartmann P.E., Geddes D.T. (2017). Changes in fatty acid composition of human milk in response to cold-like symptoms in the lactating mother and infant. Nutrients.

[80] Gorelik K.D., Gorelik Y.V., Dmitriyev A.V., Bykov K.V. (2019). Fatty acids in fat emulsions for parenteral nutrition in neonatology. Neonatol. News Opin. Train..

[81] Plotnikova E.Y., Sinkova L.K., Isakov L.K. (2018). The role of omega-3 unsaturated acids in the prevention and treatment of various diseases (part 1). Attend. Dr..

[82] Pennick G., Chavan B., Summers B., Rawlings A.V. (2012). The effect of an amphiphilic self-assembled lipid lamellar phase on the relief of dry skin. Int. J. Cosmet. Sci..

[83] Knutsen H.K., Alexander J., Barregård L., Bignami M., Brüschweiler B. (2016). Erucic acid in feed and food (англ). EFSA J..

[84] Hussain S., Rehman A.U., Luckett D.J., Naqvi S., Blanchard C.L. (2021). Protease Inhibitors Purified from the Canola Meal Extracts of Two Genetically Diverse Genotypes Exhibit Antidiabetic and Antihypertension Properties. Molecules.

[85] Mohammadzadeh S., Roohvand F., Ehsani P., Salmanian A.H., Ajdary S. (2020). Canola oilseed- and Escherichia coli-derived hepatitis C virus (HCV) core proteins adjuvanted with oil bodies, induced robust Th1-oriented immune responses in immunized mice. APMIS Acta Pathol. Microbiol. Immunol. Scand..

[86] Mandrich L., Caputo E. (2020). Brassicaceae-Derived Anticancer Agents: Towards a Green Approach to Beat Cancer. Nutrients.

[87] Wang Y., Li Y., Ruan S., Lu F., Tian W., Ma H. (2021). Antihypertensive effect of rapeseed peptides and their potential in improving the effectiveness of captopril. J. Sci. Food Agric..

[88] Forouhi N.G., Wareham N.J. (2019). Epidemiology of diabetes. Medicine.

[89] Ballantyne G.H., Wasielewski A., Saunders J.K. (2009). The Surgical Treatment of Type II Diabetes Mellitus: Changes in HOMA Insulin Resistance in the First Year Following Laparoscopic Roux-en-Y Gastric Bypass (LRYGB) and Laparoscopic Adjustable Gastric Banding (LAGB). Obes. Surg..

[90] Xu F., Mejia E.G.d., Chen H., Rebecca K., Pan M., He R., Yao Y., Wang L., Ju X. (2020). Assessment of the DPP-IV inhibitory activity of a novel octapeptide derived from rapeseed using Caco_2 cell monolayers and molecular docking analysis. J. Food Biochem..

[91] Vosough-Ghanbari S., Rahimi R., Kharabaf S., Zeinali S., Mohammadirad A., Amini S. (2010). Effects of Satureja khuzestanica on serum glucose, lipids and markers of oxidative stress in patients with type 2 diabetes mellitus: A double-blind randomized controlled trial. Evid. Based Complement. Altern. Med..

[92] Akbari F., Khodadadi S., Asgari S., Shirzad H., Mirhoseini M., Shahinfard N., Rafieian-Kopaei M. (2015). A comparative study on hypoglycemic properties, lipid profile and bioactive components of hydro-alcoholic extracts of cooked and raw Brassica napus. J. Nephropharmacol..

[93] Lorente-Cebrian S., Costa A.G., Navas-Carretero S., Zabala M., Martinez J.A., Moreno-Aliaga M.J. (2013). Role of omega-3 fatty acids in obesity, metabolic syndrome, and cardiovascular diseases: A review of the evidence. J. Physiol. Biochem..

[94] Fedor D., Kelley D.S. (2009). Prevention of insulin resistance by n-3 polyunsaturated fatty acids. Curr. Opin. Clin. Nutr. Metab. Care.

[95] Xu J., Ma C., Han L., Gao H., Zhou Q., Yang M., Chen C., Deng Q., Huang Q., Huang F. (2014). Optimized rapeseed oils rich in endogenous micronutrients ameliorate risk factors of atherosclerosis in high fat diet fed rats. Lipids Health Dis..

[96] Valdecantos M.P., Perez-Matute P., Obesity M.J.A. (2009). Oxidative stress: Role of antioxidant supplementation. Rev. Investig. Clin..

[97] Capel F., Geloen A., Vaysse C., Pineau G., Demaison L., Chardigny J.M., Michalski M.C., Malpuech-Brugère C. (2018). Rapeseed oil fortified with micronutrients can reduce glucose intolerance during a high fat challenge in rats. Nutr. Metab..

[98] Jamka M., Morawska A., Krzyżanowska-Jankowska P., Bajerska J., Przysławski J., Walkowiak J., Lisowska A. (2021). Comparison of the Effect of Amaranth Oil vs. Rapeseed Oil on Selected Atherosclerosis Markers in Overweight and Obese Subjects: A Randomized Double-Blind Cross-Over Trial. Int. J. Environ. Res. Public Health.

[99] Bowen K.J., Kris-Etherton P.M., West S.G., Fleming J.A., Connelly P.W., Lamarche B., Couture P., Jenkins D.J.A., Taylor C.G., Zahradka P. (2019). Diets enriched with conventional or high-oleic acid canola oils lower atherogenic lipids and lipoproteins compared to a diet with a western fatty acid profile in adults with central adiposity. J. Nutr..

[100] Hansson G.K. (2005). Inflammation, atherosclerosis, and coronary artery disease. N. Engl. J. Med..

[101] Seppanen-Laakso T., Laakso I., Lehtimaki T., Rontu R., Moilanen E., Solakivi T., Seppo L., Vanhanen H., Kiviranta K., Hiltunen R. (2010). Elevated plasma fibrinogen caused by inadequate alpha-linolenic acid intake can be reduced by replacing fat with canola-type rapeseed oil. Prostaglandins Leukot. Essent. Fat. Acids.

[102] Ostlund R.E., Racette S.B., Okeke A., Stenson W.F. (2002). Phytosterols that are naturally present in commercial corn oil significantly reduce cholesterol absorption in humans. Am. J. Clin. Nutr..

[103] Xu J., Zhou X., Deng Q., Huang Q., Yang J., Huang F. (2011). Rapeseed oil fortified with micronutrients reduces atherosclerosis risk factors in rats fed a high-fat diet. Lipids Health Dis..

[104] Meenakshi S., Umayaparvathi S., Arumugam M., Balasubramanian T. (2011). In vitro antioxidant properties and FTIR analysis of two seaweeds of Gulf of Mannar. Asian Pac. J. Trop. Biomed..

[105] Khatiwada B., Kunwar S., Dhakal A., Joshi A., Miya A.R., Subedi P. (2018). Total phenolic content, antioxidant activity, alpha-amylase inhibitory activity and antibacterial activity of radish seed and rapeseed. Eur. J. Biotechnol. Biosci..

[106] Szydłowska-Czerniak A., Trokowski K., Karlovits G., Szłyk E. (2010). Determination of Antioxidant Capacity, Phenolic Acids, and Fatty Acid Composition of Rapeseed Varieties. J. Agric. Food Chem..

[107] Szydłowska-Czerniak A., Łaszewska A. (2015). Effect of refining process on antioxidant capacity, total phenolics and prooxidants contents in rapeseed oils. Food Sci. Technol..

[108] Stefanucci A., Zengin G., Llorent-Martinez E.J., Dimmito M.P., della Valle A., Pieretti S., Ak G., Sinan K.I., Mollica A. (2020). Chemical characterization, antioxidant properties and enzyme inhibition of Rutabaga root’s pulp and peel (*Brassica napus* L.). Arab. J. Chem..

[109] Dusica J., Jovica V., Zorica N., Gordana P., Gordana T., Maja I., Dragana M. (2017). Antioxidant capacity of oilseed rape (Brassica napus) in different soil types. Turk. J. Agric. For..

[110] Lee N.K., Lee J.H., Lim S.M., Lee K.A., Kim Y.B., Chang P.S., Paik H.D. (2014). Short communication: Antiviral activity of subcritical water extract of Brassica juncea against influenza virus A/H1N1 in nonfat milk. J. Dairy Sci..

[111] Hajarizadeh B., Grebely J., Dore G.J. (2013). Epidemiology and natural history of HCV infection. Nat. Rev. Gastroenterol. Hepatol..

[112] Mohammadzadeh S., Roohvand F., Ajdary S., Ehsani P., Hatef Salmanian A. (2015). Heterologous Expression of Hepatitis C Virus Core Protein in Oil Seeds of *Brassica napus* L.. Jundishapur J. Microbiol..

[113] Batool N., Arshad M., Hassan F., Ilyas N., Shahzad A. (2018). Report-Physicochemical and Antimicrobial properties of canola (*Brassica napus* L.) seed oil. Pak. J. Pharm. Sci..

[114] Turgumbayeva A., Tileuberdi N., Zhakipbekov K., Tulemissov S., Umurzakhova G., Utegenova G. (2021). Antimicrobial efficacies of *Brassica Napus* L. essential oils/ nanoparticles composites. J. Nanostructures.

[115] Lee C.S., Baek J., Han S.Y. (2017). The Role of Kinase Modulators in Cellular Senescence for Use in Cancer Treatment. Molecules.

[116] Fridlender M., Kapulnik Y., Koltai H. (2015). Plant derived substances with anti-cancer activity: From folklore to practice. Front. Plant. Sci..

[117] Morrissey K.M., Yuraszeck T.M., Li C.C., Zhang Y., Kasichayanula S. (2016). Immunotherapy and Novel Combinations in Oncology: Current Landscape, Challenges, and Opportunities. Clin. Transl. Sci..

[118] Gordaliza M. (2007). Natural products as leads to anticancer drugs. Clin. Transl. Oncol..

[119] Johson I.T. (2018). Cruciferous vegetables and risk of cancer of the gastrointestinal tract. Mol. Nutr. Food Res..

[120] Arcidiacono P., Kuligina E., Crisanti A., Ragonese F., Rende M., Spaccapelo R., Stabile A., Bottoni U., Pistilli A., Calvieri S. (2018). Antitumor activity and expression profiles of genes induced by sulforaphane in human melanoma cells. Eur. J. Nutr..

[121] Bao Y., Wang W., Zhou Z., Sun C. (2014). Benefits and risks of the hormetic effects of dietary isothiocyanates on cancer prevention. PLoS ONE.

[122] Nagai T., Inoue R., Inoue H., Suzuki N. (2002). Scavenging capacities of pollen extracts from cistus ladaniferus on autoxidation, superoxide radicals, hydroxyl radicals and DPPH radicals. Nutr. Res..

[123] Steigerová J., Oklešťková J., Levková M., Rárová L., Kolář Z., Strnad M. (2010). Brassinosteroids cause cell cycle arrest and apoptosis of human breast cancer cells. Chem. Biol. Interact..

[124] Akinsete J.A., Ion G., Witteand T.R., Hardman W.E. (2012). Consumption of high ω-3 fatty acid diet suppressed prostate tumorigenesis in C3(1) Tag mice. Carcinogenesis.

[125] Takahashi S.I., Takeshita K., Seeni A., Sugiura S., Tang M., Sato S.Y., Kuriyama H., Nakadate M., Abe K., Maeno Y. (2009). Suppression of prostate cancer in a transgenic rat model via gamma-tocopherol activation of caspose signaling. Prostate.

[126] Bray F., Ferlay J., Soerjomataram I. (2018). Global Cancer Statistics 2018: GLOBOCAN Estimates of Incidence and Mortality Worldwide for 36 Cancers in 185 Countries. CA Cancer J. Clin..

[127] Bhutia S.K., Mallick S.K., Maiti T.K. (2009). In vitro immunostimulatory properties of Abrus lectins derived peptides in tumor bearing mice. Phytomedicine.

[128] Ferrero R.L., Soto-Maldonado C., Weinstein-Oppenheimer C., Cabrera-Muñoz Z., Zúñiga-Hansen M.E. (2021). Antiproliferative Rapeseed Defatted Meal Protein and Their Hydrolysates on MCF-7 Breast Cancer Cells and Human Fibroblasts. Foods.

[129] Frolova E.V. (2016). Arterial hypertension. Russ. Fam. Dr..

[130] Kearney P.M., Whelton M., Reynolds K., Muntner P., Whelton P.K., He J. (2005). Global burden of hypertension: Analysis of worldwide data. Lancet.

